# One‐Step SnO_2_ Nanotree Growth

**DOI:** 10.1002/chem.201602333

**Published:** 2016-08-17

**Authors:** Piet Schönherr, Thorsten Hesjedal

**Affiliations:** ^1^ Clarendon LaboratoryParks RoadOxfordOX1 3PUUK

**Keywords:** crystal growth, nanocatalysis, nanogrowth, nanostructures, physical vapor deposition

## Abstract

A comparison between Au, TiO_2_ and self‐catalysed growth of SnO_2_ nanostructures using chemical vapour deposition is reported. TiO_2_ enables growth of a nanonetwork of SnO_2_, whereas self‐catalysed growth results in nanoclusters. Using Au catalyst, single‐crystalline SnO_2_ nanowire trees can be grown in a one‐step process. Two types of trees are identified that differ in size, presence of a catalytic tip, and degree of branching. The growth mechanism of these nanotrees is based on branch‐splitting and self‐seeding by the catalytic tip, facilitating at least three levels of branching, namely trunk, branch and leaf.

Metal‐oxide semiconductors have served as functional materials for gas sensors for decades.^1^ The sensitivity, response speed and power efficiency scale with the size of the sensing element. Nanostructures of SnO_2_ are ideal building blocks for realising improved sensors, for example, for inflammable gases such as CO.^2^, ^3^, ^4^ Branched nanowires are particularly suited to provide high sensitivity at low cost without the need of spatial resolution, that is, it is of minor importance where a single gas molecule is detected on the sensor as long as it can be detected. The most economical method to fabricate such structures is chemical vapour transport.^5^ This method employs a catalysed growth process in which nanowires are seeded by Au nanoparticles.^6^ Branches are typically added by multi‐step catalyst seeding.^7^ Here, we demonstrate the efficient one‐step growth of SnO_2_ nanotrees using Au catalyst nanoparticles and explain the growth mechanism.

## Experimental Methods

Samples were synthesised in a horizontal tube furnace (Nabertherm B180, 25 mm‐diameter, heated length 600 mm) from Sn granules placed in a quartz boat. N_2_ at atmospheric pressure was used as a carrier gas, with a typical flow rate of 300–900 sccm. The boats were physically connected to magnetic transfer arms. The furnace was heated up to growth temperature (typically 800–1000 °C for nanowire growth) over a ramp time of one hour. Si(100) substrates were cleaved into pieces measuring 10×10 mm^2^ and then cleaned using trichloroethylene, 2‐propanol, methanol and DI water. Some of the substrates were then further functionalised with 0.1 % poly‐l‐lysine solution and coated with either a 5 nm‐diameter Au nanoparticle solution or a water‐based TiO_2_ solution, flushed with DI water, and then blow‐dried with nitrogen.^8^ They were inserted through a load‐lock with the precursor retracted to the cold zone under maximum N_2_ flow. This procedure minimises the exposure to air. The furnace was then pumped (using a membrane pump) and flushed twice for seven minutes. The precursor was subsequently moved to the centre of the furnace and the substrates 12 cm downstream. Scanning electron microscopy (SEM), energy‐dispersive X‐ray spectroscopy (EDS) and transmission electron microscopy (TEM) were used to characterise the samples. Typical growth parameters for SnO_2_ nanotrees are a N_2_ flow rate of 700 sccm and a furnace temperature of 950 °C.

## Results

**Figure 1:**
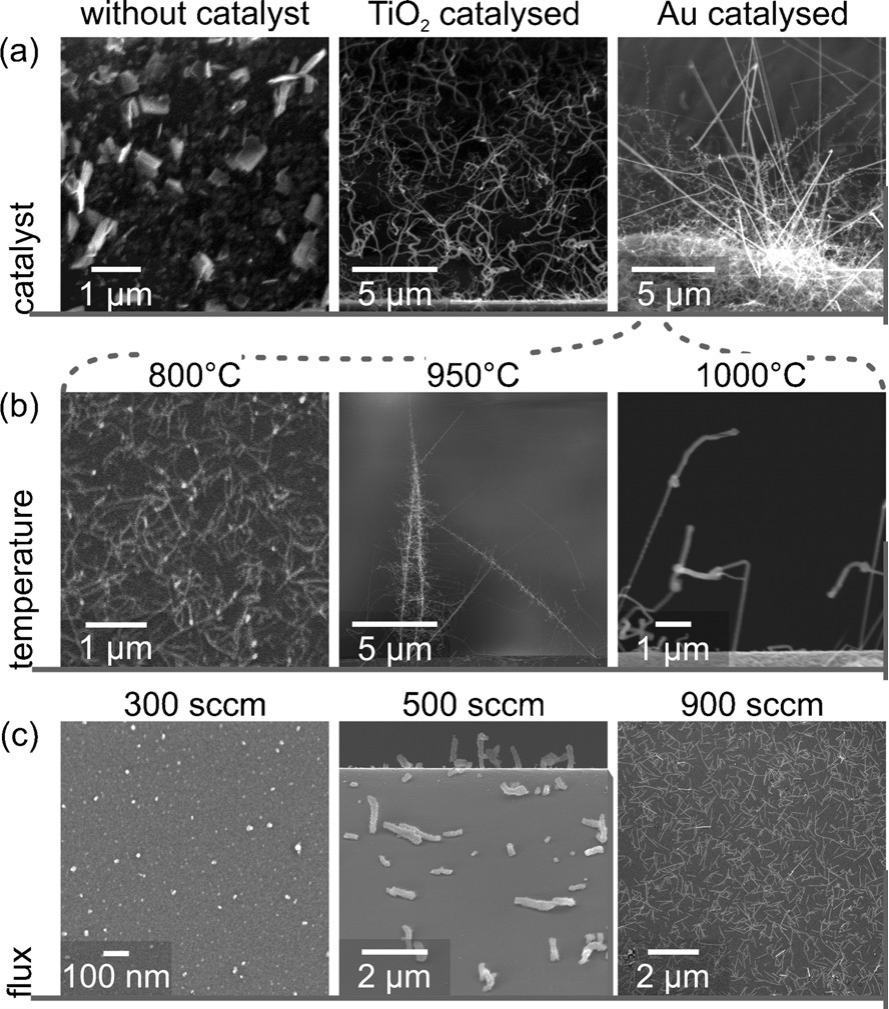
SEM images of SnO_2_ nanowire growth that characterise the impact of changing the growth parameters: catalyst, furnace temperature and flux. Three examples are displayed in each category. a) Substrate preparation using no catalyst, TiO_2_ nanoparticles and Au nanoparticles (left to right); b) Furnace temperature increased to 800, 950 and 1000 °C. c) Flux‐dependence of SnO_2_ nanowire growth at 300, 500 (grey sideline indicates substrate edge) and 900 sccm.

The parameter space of SnO_2_ nanowire growth was studied systematically using a fast‐load‐lock CVD system. Figure 1 shows the results of three sets of growth experiments varying the catalyst, furnace temperature and flux ( Figure 1 a–c). Three samples were prepared for the catalyst comparison shown in Figure 1 a: without catalyst, with 20 nm‐diameter TiO_2_ nanoparticles (rutile and anatase mixture P‐25)^8^ and with 5 nm‐diameter Au nanoparticles. Recently, TiO_2_ nanoparticles were demonstrated to outperform Au as a catalyst for Bi_2_Se_3_ nanowire growth.^8^ Only plates grow from the substrate without catalyst, whereas both TiO_2_ and Au enable nanowire growth. TiO_2_‐grown nanowires form an intertwined, free‐standing network. Au leads to a homogeneous coverage with straight nanowires (diameter :50 nm) and nanostars, as shown in Figure 1 a. Therefore, we used Au nanoparticles in all subsequent growth experiments. The temperature window for nanowire growth between 800 and 1000 °C is depicted in Figure 1 b. In principle, Sn vapour transport is possible at lower temperatures because we found occasional 20 μm‐sized clusters down to 600 °C. The highest yield of nanowires was achieved at temperatures around 800 °C. At 1000 °C, the nanowires have a poor morphology, as determined by SEM. At around 950 °C, the formation of branches from very long nanowires dominates the growth (c.f. Figure 1 b, centre). These nanotrees lie flat on the substrate or grow free‐standing from the sides of the substrate. A flux of 700 sccm was used for the temperature series. The high flux is crucial for SnO_2_ nanowire growth, as can be seen in Figure 1 c. The catalyst particles only accumulate material and grow into spheres at too low flux but nucleation is not initiated owing to limited precursor supply. There is a threshold between 300 and 500 sccm at 800 °C furnace temperature where nucleation starts to occur. The large diameter of the nanowires grown at 500 sccm means that the axial growth rate is very slow and that radial growth dominates. It is the supersaturation of the catalytic tip that drives the growth at high enough flux (1 sccm). The resulting nanowires are thin and long. Many catalyst sites can be activated such that the substrate coverage, that is, the yield, is very high.

All nanowires that were grown using the Au catalyst have a catalytic tip larger than the 5 nm diameter of the initial Au nanoparticle. This is evidence of the fact that the catalyst forms an alloy with incoming precursor vapour and grows in size until supersaturation is reached, consistent with the vapour–liquid–solid (VLS) model.

TiO_2_, however, is not liquid during the growth because of its higher melting point, so the VLS mechanism can be ruled out. It either forms a solid solution with SnO_2_ or facilitates the formation of Sn droplets that allow for self‐catalysed growth. TiO_2_ catalyst particles should be found at the tip if a solid solution was formed to lead to tip‐catalysed growth. TEM EDS does not detect any TiO_2_ in the tip of the nanowire. Therefore, it can be concluded that the TiO_2_ nanoparticles are located at the base of the wire. Growth is initiated by the formation of Sn droplets on their surface. Figure 1 a shows such droplets as dark spots on TiO_2_ patches. The nanowires are amorphous.

**Figure 2:**
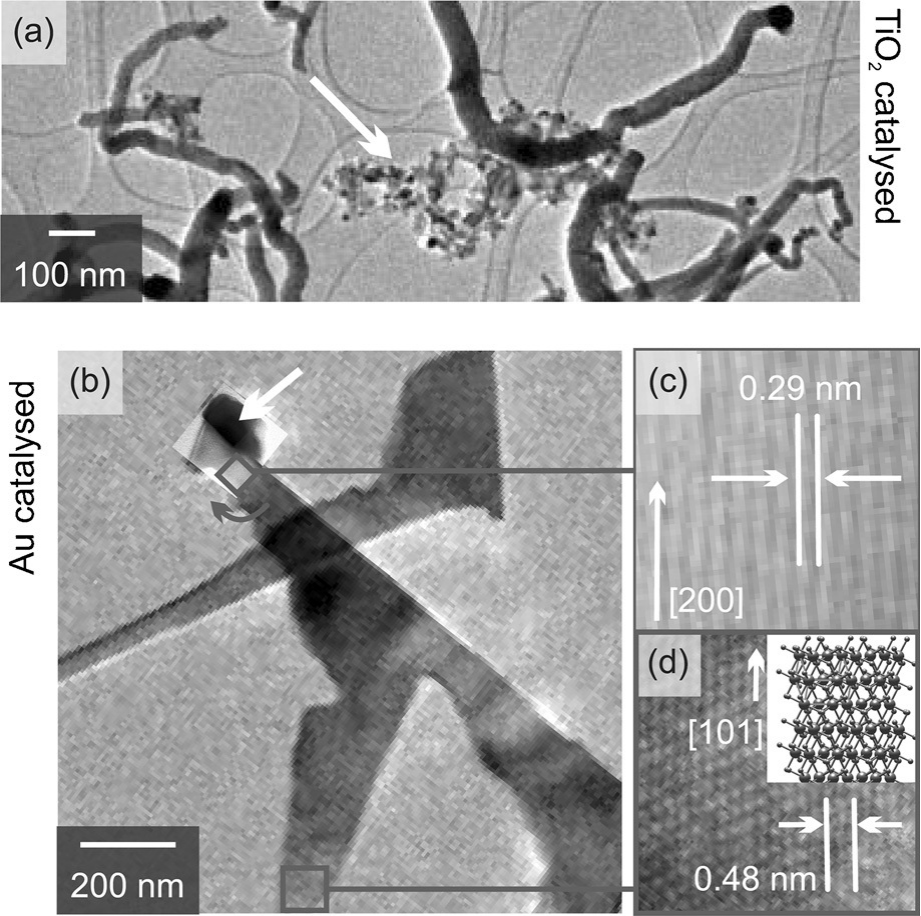
TEM images of SnO_2_ nanowires. a) TiO_2_ catalysed growth: The catalyst particles remain at the root of the nanowires. TiO_2_ patches facilitate the formation of droplets that appear as dark spots (white arrow). b) Au nanoparticle catalyst: A catalytic particle is formed at the tip of the nanowires (white arrow). Two types of crystal structures are observed. c) β‐Sn with a lattice spacing of 0.29 nm consistent with the [200] growth direction (rotated by 46° with respect to (b)); (d) tetragonal SnO_2_ with a lattice spacing of 0.48 nm. A sketch of the lattice illustrates the unit cell (blue) and the [101] growth direction.

Au‐catalysed nanowires grow longer and straight compared to the TiO_2_ catalysed nanowires (c.f. Figure 2 b) and branch occasionally, as also reported by Jin et al. for catalyst‐free growth.^5^ In the centre of the catalytic tip is a dark spot, which is roughly the size of a single Au nanoparticle or a cluster of several nanoparticles, indicative of VLS growth. Whereas most of the nanostructures are composed of SnO_2_, a small fraction of pure Sn is also found (see high resolution image of the tip area in Figure 2 c). The growth direction of this short wire is [200], consistent with Sn nanowire growth.^9^ The SnO_2_ nanowires have a tetragonal crystal structure and grow along the [101]‐direction, as shown in Figure 2 d.

**Figure 3:**
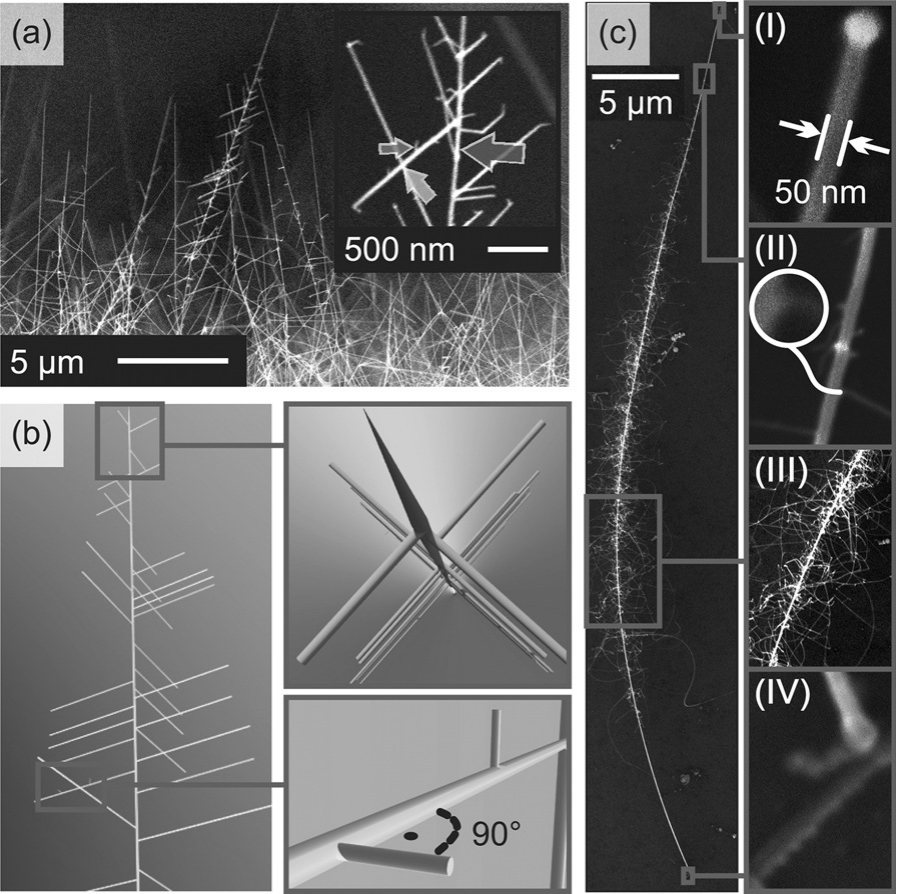
SEM images of SnO_2_ nanotrees. a) Vertical trees at the substrate edge. The inset shows a trunk (brown arrow) with branches (yellow arrow) and leaves (green arrow). b) Sketch of a nanowire tree. The tip is tapered with no catalyst particle visible at the top (upper inset). Both trunk and branches, as well as branches and leaves are perpendicular to each other (lower inset). c) Horizontally oriented nanotree on the surface. There are four sections (I–IV) with distinctive features: tip (I), little branch growth with extrusion in the inset (II), middle of the trunk (III) and root (IV).

Growth at 950 °C and a flux of 700 sccm lead to trees with and without catalytic tips. A nanowire tree without catalytic tip is tapered, as shown in Figure 3 a and b. It consists of three components: trunk, branches and leaves. The components grow perpendicular to each other and become gradually smaller. Branches at the bottom are longer than branches at the top. The nanotree in Figure 3 c is larger and consists of a trunk with 50 nm diameter and fine branches with 30 nm diameter. The branches are curled up and have grown denser than the tree in Figure 3 a.

The four sections of the trunk are described in the following. The tip is formed by a droplet as usually observed for VLS‐grown nanowires. The next section is marked by the appearance of short branches. The number and length of branches increases towards the main section. Branches are connected to the tree by extruding bases (Figure 3 c (II), inset). The fourth section near the base is completely free of branches. An amorphous structure on the root indicates the first tens of nanometres where the growth started.4

**Figure 4:**
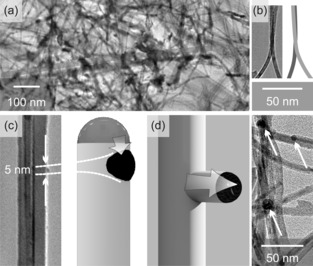
Formation mechanism of SnO_2_ nanotree branches. a) TEM image of a nanotree structure. The trunk is indicated by a red line. b) Splitting of a branch. c) 5 nm‐sized particle on the side‐wall of a branch. d) Sketch of the initial stages of the branch growth and observation of catalyst tips on branches (arrows).

What is the mechanism for the formation of branches? A low magnification TEM image in Figure 4 a gives an overview over the density and length of the branches. The density is especially increased by separation. In Figure 4 b a nanobranch splits in the centre and separates into two branches. This can be induced by a crystal defect or, more likely, a split catalyst particle resulting in an additional growth direction. High magnification images reveal 5 nm‐sized particles on the walls of the trunk, as shown in Figure 4 c. The size is identical to the particle size of our commercial Au nanoparticles. There are three scenarios that can explain this feature: 1) Au nanoparticles migrate over the SnO_2_ surface and deposit onto the trunk. The 5 nm particle shown in Figure 4 c could be such a nanoparticle. However, this would require an exceptional mobility of Au on SnO_2_ at this temperature; 2) Sn precursor atoms spontaneously form droplets on the SnO_2_ trunk and branches grow self‐catalysed following the VLS mechanism. This also explains the observation in Figure 4 c and the formation of leaves grown from smaller droplets on the branches; 3) the main catalyst particle at the tip gives off smaller particles if the size exceeds a certain threshold,^10^ as depicted in Figure 4 c. These particles can even migrate several micrometers along the trunk.^11^ During the first few micrometers of growth the size does not exceed the threshold yet. Hence, fewer branches grow at the bottom. Branch length is a function of height, measured from the trunk, with the longest branches towards the root and short branches, or even seeds, at the top. Leaves grow also mediated by catalyst migration on branches. Some of the nanotrees do not have a catalyst particle attached to the tip, and it can be assumed that it has been consumed during growth, yet they still grow branches.

In conclusion, Au and TiO_2_ nanoparticles are efficient catalysts for SnO_2_ nanowire growth. Networks of polycrystalline and bent or single‐crystalline, straight and branched nanowires can be grown by tuning the growth parameters. TiO_2_ catalyst particles facilitate the self‐catalysed growth by enabling Sn droplet formation on the catalyst surface. At 950 °C Au nanoparticles diffuse over the substrate and the grown structures. This leads to a preferential growth on nanowires forming trunks, branches and leaves. These nanotrees are excellent building blocks for highly‐sensitive gas detectors and other nanoelectronic devices.

## References

[1] N. Yamazoe , Sens. Actuators B 1991, 5, 7–19.

[2] A. Kolmakov , Y. Zhang , G. Cheng , M. Moskovits , Adv. Mater. 2003, 15, 997–1000.

[3] X. Chen , C. K. Y. Wong , C. A. Yuan , G. Zhang , Sens. Actuators B 2013, 177, 178–195.

[4] H. Feng , J. Huang , J. Li , Chem. Commun. 2013, 49, 1017–1019. 10.1039/c2cc38463a 23258304

[5] C. Jin , S. Park , C.-W. Kim , M.-S. Choi , C. Lee , D. Lee , Mater. Lett. 2015, 158, 5–8.

[6] J. X. Wang , D. F. Liu , X. Q. Yan , H. J. Yuan , L. J. Ci , Z. P. Zhou , Y. Gao , L. Song , L. F. Liu , W. Y. Zhou , G. Wang , S. S. Xie , Solid State Commun. 2004, 130, 89–94.

[7] D. Wang , F. Qian , C. Yang , Z. Zhong , C. M. Lieber , Nano Lett. 2004, 4, 871–874.

[8] P. Schönherr , D. Prabhakaran , W. Jones , N. Dimitratos , M. Bowker , T. Hesjedal , Appl. Phys. Lett. 2014, 104, 253103.

[9] Y.-J. Hsu , S.-Y. Lu , Y.-F. Lin , Small 2006, 2, 268–273. 17193034 10.1002/smll.200500303

[10] C.-Y. Nam, D. Tham, J. E. Fischer in *MRS Proceedings*, Cambridge Univ. Press, Cambridge, **2007**, *1058*, 1058-JJ04-03, DOI: 10.1557/PROC-1058-JJ04-03.

[11] T. Kawashima , T. Mizutani , T. Nakagawa , H. Torii , T. Saitoh , K. Komori , M. Fujii , Nano Lett. 2008, 8, 362–368. 18095731 10.1021/nl072366g

